# Maternal satisfaction towards childbirth Service in Public Health Facilities at Adama town, Ethiopia

**DOI:** 10.1186/s12978-020-00911-0

**Published:** 2020-05-06

**Authors:** Meron Tadele, Dereje Bikila, Robera Olana Fite, Mohammed Suleiman Obsa

**Affiliations:** 1 Department of Maternity and Reproductive Health Nursing, Adama General Hospital and Medical College, Adama, Ethiopia; 2 Department of Clinical Nursing, College of Medicine and Health Sciences, Arsi University, Asella, Arsi zone, Oromia Ethiopia; 3 Department of Nursing, College of Health Sciences and Medicine, Wolaita Sodo University, Po-Box: 138, Wolaita Sodo, Ethiopia; 4 Department of Anesthesia, College of Health Sciences and Medicine, Wolaita Sodo University, Wolaita Sodo, Ethiopia

**Keywords:** Satisfaction, Maternal, Childbirth

## Abstract

**Background:**

Maternal satisfaction towards childbirth service is related to the quality of care. Promotion of patient satisfaction is essential for preventing patient anxiety, promoting treatment adherence, preventing disease, and health promotion. This study was aimed at assessing the satisfaction and associated factors among mothers who visit public health facilities in Adama town for childbirth service.

**Methods:**

An institution based cross-sectional study design was conducted at public health facilities in Adama town from June 01 to June 30, 2018. Four hundred seventy-seven mothers were selected using a systematic random sampling method. Bivariate and multivariate logistic regressions were conducted to identify predictors of maternal satisfaction towards childbirth service by considering *p*-value less than 0.05.

**Results:**

The study revealed that 357 (74.8%) were satisfied with the services. Factors which showed a significant association with satisfaction were 25–34 age group (AOR; 2.026, 95%CI:1.056,3.887), no formal education (AOR;2.810, 95%CI;1.085,7.278), planned childbirth (AOR; 1.823,95%CI;1.024,3.246), wait time of less than 1 h (AOR;11.620,95%CI;3.619,37.309) and wait time of one to 2 h (AOR;19.620, 95%CI;2.349,68.500).

**Conclusion:**

Three-quarters of the mothers were satisfied with childbirth services. Age, educational status, reason for visit and wait time were found to have a significant association with maternal satisfaction of childbirth services.

## Plain English summary

Ensuring maternal satisfaction of childbirth services is essential for preventing anxiety, promoting treatment adherence, preventing disease and health promotion. In order to improve future use of maternal health services, health facilities should focus on improving the quality of care. This study assessed the satisfaction and associated factors among mothers who visited public health facilities in Adama town, Ethiopia for childbirth services. An institution based cross-sectional study design was used from June 01–30, 2018. Four hundred seventy-seven mothers participated. Three-quarters of the mothers were satisfied with the childbirth service. Age, educational status, reason for visit and wait time were found to have a significant association with satisfaction of services. The results suggest intervention is required to improve the quality of service.

## Introduction

Client satisfaction of childbirth services is related to the quality of care [1]. Satisfaction is a means of preventing maternal death related to the lack of adherence to health counseling [1, 2]. Increasing satisfaction of services has long-term benefit for both the community and patients. If high quality care is provided, the utilization of reproductive and sexual health services will increase [3, 4].

Health service satisfaction is influenced by cultural beliefs, accessibility, socioeconomic status, affordability, health professionals’ approach, cleanliness of wards, and waiting rooms [4].

The health of the whole community is directly linked to the health of reproductive age women. There is a relationship between maternal and neonatal health. The immunization status, childhood development, and survival are affected by maternal health status. Healthier women and children create a better society [5–7]. Therefore, effective basic antenatal care, skilled birth attendance, post-partum care, and treatment of complications is essential [8, 9]. Therefore, to decrease the maternal and child mortality and morbidity, affordable, respectful and evidence-based care is necessary [10].

Satisfaction of services reflects expectations and the quality of health care received [11]. Every health professionals should work skillfully and practice with compassionate care [12, 13].

The World Health Organization (WHO) recommends respectful, women-centered and evidence-based maternity practice, which improves birth outcomes. Furthermore, maintaining the highest standard of personal conduct, integrity, proper and effective communication with patients is an integral component of the health system. Therefore, incorporating these principles may improve outcomes [14, 15].

In developing countries, still, less than 50% of the mothers are satisfied with the childbirth services [3]. In urban areas of Ethiopia, the institutional delivery rate is 79%. According to the Ethiopian Ministry of Health sector transformation plan, maternal and newborn health is a priority. Respect to patient’s autonomy, dignity, feelings, and preferences is mandatory. To increase the institutional childbirth rate and promote the utilization of postnatal care, care should focus on maintaining client satisfaction [16].

Assessing satisfaction of childbirth services and health care facilities might help to guide the development and improvement of services; one way of doing this is by surveying patients who have used health services. This study tried to assess maternal satisfaction and associated factors in three public health facilities at Adama town. This information will be helpful for the health professional to improve the quality of service delivery. In addition, the study findings can help health policymakers and other sectors working on maternal health to standardize the existing interventions.

## Methods

### Study area, period and design

A cross-sectional study was conducted at Adama hospital and medical college, Adama health center and Geda health center in Adama, East Shewa Zone, Ethiopia. The study was conducted from June 01 to June 30, 2018.

### Population

#### Inclusion and exclusion criteria

All mothers who visited the public health hospital at Adama town for childbirth services were included, those who were critically ill were excluded.

### Sample size and sampling procedure

The required sample size was determined by using a single population proportion formula with the following assumptions, 74.9% maternal satisfaction level [17] with 95% confidence interval and 4% margin of error. Adding of 10% non-response rate, the final sample size was 497.

One governmental hospital and two health centers were selected using a random sampling method, and then the sample was proportionally allocated among the health institutions. Systematic random sampling was used to select participants,. Every fourth delivering mother in the governmental hospital was included and every third delivering mother was selected in each health center.

### Operational definition

#### Satisfaction

Those who reported being satisfied with greater or equal to 75% of items were categorized as satisfied, while those who were satisfied in less than 75% of the items were categorized as unsatisfied [8–12, 17, 18].

### Data collection instrument and procedure

The structured questionnaire was adapted from the Donabedian quality assessment framework [18] and presented using a 5-scale likert scale (1-very dissatisfied, 2-dissatisfied, 3-neutral, 4-satisfied, and 5-very satisfied). The questionnaire was shown to be reliable in the study (Cronbach’s α = 0.725). The questionnaire sought information on the socio-demographic characteristics, obstetrics history, service characteristics and satisfaction level (Additional file 2).

Data collection was carried out in the postpartum unit. Four midwifery nurses were recruited as data collectors. One supervisor monitored the data collection. Training was provided for the data collectors and the supervisor for 2 days by the principal investigator. The training included the study objective, the meaning of each question and interviewing technique.

### Data quality assurance

After completing training, data collection was piloted. During the data collection period, completeness, consistency, and accuracy of each questionnaire were reviewed. Corrective measures were taken, when necessary by the research team, to ensure a common understanding of each question. A translator was also used in some interviews.

### Data processing and analysis

All data was checked manually for completeness and then coded and entered using EpiData version 3.1. Data were exported to SPSS version 20. Chi-Square was used to test a significant difference of satisfaction level among mothers who delivered in the three public health facilities. Significance was determined by using crude and adjusted odds ratios with 95% confidence intervals. To assess the association between the dependent variables and independent variables, bivariate logistic regression was used. Then multivariable logistic regression was employed to identify different predictors by considering *p*-value less than 0.05.

## Results

### Socio-demographic characteristics

A total of 477 women participated in the study obtaining a response rate of 96%. Of the respondents, 240(50.3%) were 25–34 years of age. Three hundred eighty-eight (81.3%) were married. Three hundred eighty-one (79.9%) attended secondary school or greater. Two hundred ten (44%) of them belong to the Oromo ethnic group and 182(10.3%) were Muslim. One hundred seven (22.4%) were government employees and 410(86%) lived in urban areas. The average monthly salary was 2265.13 Ethiopian Birr (Table 1).

**Table 1:** Socio-demographic characteristics of the mothers in public health facilities at Adama town, Ethiopia, June 01–30, 2018

Variable	Frequency	Percentage
**Age**		
15–24	171	35.8
25–34	240	50.3
35 and above	66	13.8
**Marital status**		
Married	75	15.7
Single	388	81.3
Divorced	4	0.8
Widowed	10	2.1
**Educational status**		
No formal education	48	10.1
Primary	48	10.1
Secondary and above	381	79.9
**Ethnicity**		
Amhara	108	22.6
Oromo	210	44
Tigrie	45	9.4
Gurage	66	13.8
Others	48	10.1
**Religion**		
Orthodox	147	9.4
Muslim	182	10.3
Protestant	120	39.2
Others	28	41.1
**Occupation**		
Housewife	104	21.8
Farmer	47	9.9
Merchant	97	20.3
Government Employee	107	22.4
Others	122	25.6
**Residence**		
Urban	410	86
Rural	67	14
**Monthly salary**^a^		
< 1000	94	19.7
1001–2000	163	34.2
2001–3000	142	29.8
3001–4000	71	14.9
> 4001	7	1.5

^a^: Ethiopian Birr (1 Ethiopian Birr = 0.031 United States Dollar)

### Obstetrics history

One hundred eighty-five (38.8%) participants had parity of two. Regarding the reason for visit, 334(70%) of the mothers had a planned childbirth. The majority (72.1%) of women reported an unwanted pregnancy. One hundred ninety-seven (41.1%) delivered by cesarean section. Four hundred nineteen (87.8%) women did not have a delivery-related complication. Four hundred twenty-five (89.1%) women had a live born. Four hundred twenty-seven (89.5%) arrived to facility by car. The majority (93.1%) of the mothers attended antenatal care (ANC) and 354(74.2%) delivered in a health institution. One hundred twenty-seven (26.6%) were referred from another health institution (Table 2).

**Table 2:** Obstetrics history of the mothers in public health facilities at Adama town, Ethiopia, June 01–30, 2018

Variable	Frequency	Percentage
**Parity**		
One	162	34
Two	185	38.8
Three and above	130	27.2
**Reason for visit**		
Planned childbirth	334	70
Referral for childbirth	143	30
**Pregnancy status**		
Unwanted	344	72.1
Wanted	133	27.9
**Mode of childbirth**		
Spontaneous vaginal delivery	191	40
Assisted delivery	89	18.7
Cesarean section	197	41.1
**Delivery related complications**		
Yes	58	12.2
No	419	87.8
**Fetal Outcome**		
Live	425	89.1
Dead	52	10.9
**Mode of transportation**		
Car	427	89.5
On foot, animal, carried by human	50	10.5
**ANC follow-up**		
Yes	444	93.1
No	33	6.9
**Previous childbirth at health institution**		
Yes	354	74.2
No	123	25.8
**Referred from another health institution**		
Yes	127	26.6
No	350	73.4

### Satisfaction of services

Of the respondents, 162(34%), 385(80.7%), 436(91.4%), were satisfied by the health facility in regards to distance, information service, and wait time (Table 3).

**Table 3:** Satisfaction level of the service characteristics in public health facilities at Adama town, Ethiopia, June 01–30, 2018 June 01–30, 2018

Variables	Satisfaction	
	Yes	No
	Frequency(percentage)	Frequency(percentage)
**Health facility distance**	162 (34)	315 (66)
**Information service**	385 (80.7)	92 (19.3)
**Toilet cleanliness and access**	131 (27.5)	346 (72.5)
**Complete information provision**	143 (30)	334 (70)
**Cost paid**	71 (14.9)	406 (85.1)
**Confidentiality of provider**	266 (55.8)	211 (44.2)
**Drug and supplies availability**	294 (61.6)	183 (38.4)
**Privacy**	268 (56.2)	209 (43.8)
**Respect and courtesy of staff**	192 (40.3)	285 (59.7)
**Examination room cleanliness**	181 (37.9)	296 (62.1)
**Waiting area cleanliness**	185 (38.8)	292 (61.2)
**Wait time**	436 (91.4)	41 (8.6)
**Overall cleanliness of facility**	138 (28.9)	339 (71.1)

### Characteristics of the service delivered

461(96.6%) reported that the facility had no waiting area. Three hundred fifty-two (73.8%) had reported that the wait time was less than 1 h. Two hundred ninety-six (62.1%) received services from midwives. Two hundred eight-seven (60.2%) of them received services from male health professionals. Three hundred sixty-six (76.7%) and 393(82.4%) would like to use institutional delivery in the future and recommend institutional delivery to others, respectively (Table 4).

**Table 4:** Characteristics of the service delivered in public health facilities at Adama town, Ethiopia, June 01–30, 2018

Variables	Frequency	Percentage
**Waiting area**		
Yes	16	3.4
No	461	96.6
**Wait time**		
Less than 1 h	352	73.8
1–2 h	99	20.8
More than 2 h	26	5.5
**Health professional**		
Doctor	181	37.9
Midwife	296	62.1
**Sex of the health professional**		
Male	287	60.2
Female	190	39.8
**Future use of institutional delivery**		
Yes	111	23.3
No	366	76.7
**Recommend institutional delivery to others**		
Yes	84	17.6
No	393	82.4

### Level of satisfaction of childbirth service

**Fig. 1:**
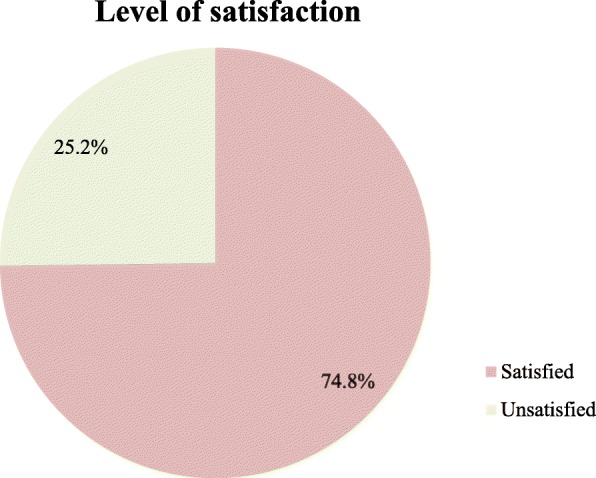
Maternal satisfaction towards childbirth service in public health facilities at Adama town, Ethiopia, June 01–30, 2018

Of the respondents, 357 (74.8%) and 120 (25.2%) were satisfied and unsatisfied by the services, respectively (Fig. 1). Furthermore, a significant difference was observed regarding the level of satisfaction among mothers who delivered at Adama hospital, Adama health center and Geda health center (Table 5).

**Table 5:** Maternal satisfaction towards childbirth service among mothers who delivered at Adama hospital, Adama health center and Geda health center, Ethiopia, June 01–30, 2018

Variable	Adama hospital	Adama health center	Geda health center	X^2^	*P*-value
	Frequency (Percentage)	Frequency (Percentage)	Frequency (Percentage)		
**Satisfaction level**					
**Satisfied**	195 (62.3)	108 (99.1)	54 (98.2)	76.078	< 0.01
**Unsatisfied**	118 (37.7)	1 (0.9)	1 (1.8)		

### Factors associated with the satisfaction on childbirth service

Age, educational status, residence, pregnancy status, mode of delivery, reason for visit and wait time were included in the final model for satisfaction of childbirth services. The multivariable logistic regression revealed that women 25–34 years (AOR; 2.026, 95%CI: 1.056, 3.887), with no formal education (AOR; 2.810, 95%CI: 1.085, 7.278), with a planned childbirth (AOR; 1.823, 95%CI: 1.024, 3.246), who encountered wait times under 1 h (AOR; 11.620, 95%CI: 3.619, 37.309) and wait times of one to 2 h (AOR; 19.620, 95%CI: 2.349, 68.500) had increased likelihood of being satisfied with childbirth services (Table 6).

**Table 6:** Factors associated with maternal satisfaction towards childbirth service in public health facilities at Adama town, Ethiopia, June 01–30, 2018

Variables	Satisfaction level		COR(95% CI)	AOR(95% CI)
	Satisfied	Unsatisfied		
**Age**				
15–24	121	50	1.383 (0.759,2.520)	1.183 (0.605,2.313)
25–34	194	46	2.410 (1.328,4.372)*	2.026 (1.056,3.887)*
35 and above	42	24	1	1
**Educational status**				
No formal education	38	10	1.371 (0.659,2.852)	2.810 (1.085,7.278)*
Primary	39	9	1.563 (0.731,3.341)	1.376 (0.619,3.058)
Secondary and above	280	101	1	1
**Residence**				
Urban	309	11	1.211 (0.680,2.156)	1.127 (0.515,2.465)
Rural	48	19	1	1
**Pregnancy status**				
Unwanted	270	74	1.929 (1.242,2.996)*	1.215 (0.712,2.073)
Wanted	87	46	1	1
**Mode of childbirth**				
Spontaneous vaginal delivery	147	44	1.360 (0.862,2.147)	1.125 (0.646,1.961)
Assisted delivery	70	19	1.500 (0.829,2.715)	1.754 (0.879,3.501)
Cesarean section	140	57	1	1
**Reason for visit**				
Planned childbirth	255	79	2.550 (1.667,3.899)**	1.823 (1.024,3.246)*
Referral for childbirth	102	41	1	1
**Wait time**				
Less than 1 h	269	83	17.825 (5.973,53.198)**	11.620 (3.619,37.309)**
1–2 h	84	15	30.800 (9.289,102.123)**	19.142 (5.349,68.500)**
More than 2 h	4	22	1	1

*: Significant association at *p*-value < 0.05, **: Significant association at *p*-value < 0.001

## Discussion

In this study, 74.8% were satisfied with the care delivered during childbirth. This finding is similar to a study conducted in Bahir Dar, which stated that 74.9% of the mothers were satisfied with their care [17]. This is lower than a report from studies conducted in Northwest Ethiopia, Southwest Ethiopia, Oromia region, west Gojjam, mid-western Nepal, and Gamo Gofa which stated that 81.7, 79.1, 80.7, 88, 89.88, 90.2% of the mothers were satisfied, respectively [19–24]. On the other hand, the finding is higher than a report from studies conducted in Addis Ababa, Amhara region, Jimma zone, and Eritrea, which stated that 19, 61.9, 65.2, and 20.8% of mothers were satisfied, respectively [25–28]. The differences might be related to the quality of services delivered at various health institutions and differing expectations. Furthermore, differences might be related to the study settings, study period or type of health institution. Dissatisfaction might be related to the fact that most women in this study had an unwanted pregnancy. In addition, the majority of the health professionals were male; however, in developing countries, most women prefer to have a care provided by women [3].

In this study, having no formal education had a positive association with satisfaction towards childbirth services. This is supported by studies conducted in mid-western Nepal [23], Gamo Gofa [24], and Ethiopia [29]. The expectation of services may be related to levels of knowledge [30]. Knowledge of the mothers is influenced by educational status [31].

Admission for a planned delivery was positively associated with satisfaction of childbirth services.

Wait times of less than 1 h or one to 2 h were positively associated with satisfaction of childbirth services. Research in South Ethiopia [24], Gamo Gofa [20] and Kenya [32] showed that mothers with less hours of waiting time had an increased satisfaction towards the childbirth service.

Mothers aged 25–34 years were two times more likely to be satisfied with childbirth services compared to those aged 35 and older. This is supported by research conducted in Jimma zone [27] and Oromia region [21]. This finding is opposite to a report from a study conducted in Bahirdar, which showed that women aged 20 to 34 years were less likely to satisfy with the care received compared to women aged 35 to 49 years [18]. In this study, most of the mothers aged 25–34 years had a complication as compared to those aged 35 and above. This might create dissatisfaction among the mothers aged 25 to 34.

The strength of the study was the involvement of three health facilities. It also used standardized measurement scale. It has to be noted that the finding of this study mainly reflects the situation in Adama town. Therefore, the findings should be interpreted with caution. The responses might be subject to social desirability bias. The factors expected to influence satisfaction of childbirth services may not be exhaustive.

## Conclusion

Only three-quarters of the mothers were satisfied with the childbirth service. Age, educational status, reason for visit and wait time were found to have a significant association with the satisfaction services. This study was not able to address the perception of health professionals and this could be an area of future research. In addition, future qualitative and quantitative research could explore the mechanism required to promote the quality of childbirth services.

## Supplementary information

**Additional file 1.** Conceptual Framework

**Additional file 2.** Questionnaire

## Data Availability

The data-sets used and analyzed during the current study are available from the corresponding author on reasonable request.

## Abbreviations

ANC: Antenatal Care
AOR: Adjusted Odds Ratio
CI: Confidence Interval
COR: Crude Odds Ratio
