# Supplementary material for: Maternal satisfaction towards childbirth Service in Public Health Facilities at Adama town, Ethiopia
Source: Reprod Health. 2020 May 6;17:60. doi: 10.1186/s12978-020-00911-0 (PMC7201691; doi:10.1186/s12978-020-00911-0)
Supplement: Supplementary file 1 — Additional file 1. Conceptual Framework [file 12978_2020_911_MOESM1_ESM.docx]

**Conceptual Framework**

**Obstetrics factors**

Reason for visit

Pregnancy status

Mode of delivery

Maternal Outcome

Fetal Outcome

Antenatal Care follow-up

Previous delivery at health institution

Referral from other health institution

**Socio - demographic factors**

Age

Marital status

Educational status

Occupation

Residence

Monthly Income

**Maternal Satisfaction towards Childbirth Service**

**Service related factors**

Waiting area

Waiting time

Sex of the health professional
