# Supplementary material for: Maternal satisfaction towards childbirth Service in Public Health Facilities at Adama town, Ethiopia
Source: Reprod Health. 2020 May 6;17:60. doi: 10.1186/s12978-020-00911-0 (PMC7201691; doi:10.1186/s12978-020-00911-0)
Supplement: Supplementary file 2 — Additional file 2. Questionnaire [file 12978_2020_911_MOESM2_ESM.docx]

## Questionnaire

A Questionnaire for Data Collection on Maternal Satisfaction towards Childbirth Service in Public Health Facilities at Adama town, Ethiopia

**Information sheet**

Hello, my name is _________________. I would like to interview you a few questions regarding the Childbirth Service in Public Health Facilities at Adama town, Ethiopia. Your cooperation and willingness for the interview is helpful in identifying problems related to the subject matter. The interview will take about 30 minutes. Your name will not be written in this form. All information that you give will be kept strictly confidential. Your participation is voluntary and you are not obliged to answer any question you do not wish to answer. If you feel discomfort with the interview please feel free to drop it any time you want. Do you have any questions on what we talked so far?

**Verbal consent form**

Now, do you agree to participate?

Yes __________ No ____________ If no, respect the decision and thank her. If yes continue the interview.

Interviewer name ______________________ signature ______ Date______

Supervisor name ______________________ signature ______ Date ______

**Appendix 2: Questionnaire**

**Instructions for data collectors:**

1. **Encircle the response of the respondent**
2. **Please write the response for questions which require additional information**

| **Part 1: Socio-demographic characteristics** | |
| --- | --- |
| Variable | **Response** |
| Age | _______________________ years |
| Marital status | 1. Married 2. Single 3. Divorced 4. Widowed |
| Educational status | 1. No formal education 2. Primary 3. Secondary and above |
| Ethnicity | 1. Amhara 2. Oromo 3. Tigrie 4. Gurage 5. Others, specify ______________ |
| Religion | 1. Orthodox 2. Muslim 3. Protestant 4. Others , specify ______________ |
| Occupation | 1. Housewife 2. Farmer 3. Merchant 4. Government Worker 5. Others , specify ____________ |
| Residence | 1. Urban 2. Rural |
| Monthly Income | _____________________ |
| **Part 2: Obstetrics history and service characteristic** | |
| Parity | _______________________ |
| Reason for visit | 1. Planned childbirth 2. Referral for childbirth |
| Pregnancy status | 1. Unwanted 2. Wanted |
| Mode of Childbirth | 1. Spontaneous Vaginal Delivery 2. Assisted delivery 3. Cesarean Section |
| Maternal Outcome | 1. Normal 2. Complicated |
| Fetal Outcome | 1. Live 2. Dead |
| Mode of transportation | 1. Car 2. On foot, animal, carried by human |
| ANC follow-up | 1. Yes 2. No |
| Previous childbirth at health institution | 1. Yes 2. No |
| Referred from other health institution | 1. Yes 2. No |
| Waiting area | 1. Yes 2. No |
| Waiting time | _____________ Hours |
| Health professional | 1. Doctor 2. Midwife |
| Sex of the health professional | 1. Male 2. Female |
| Use in future | 1. Yes 2. No |
| Recommend to others | 1. Yes 2. No |

| **Part 3: Satisfaction level** | | | | | |
| --- | --- | --- | --- | --- | --- |
| **1= Strongly Disagree**  **2= Disagree**  **3= Neutral**  **4= Agree**  **5= Strongly agree** | | | | | |
| Variables | Response | | | | |
| Health facility Distance | 1 | 2 | 3 | 4 | 5 |
| Information service | 1 | 2 | 3 | 4 | 5 |
| Toilet cleanliness and access | 1 | 2 | 3 | 4 | 5 |
| Complete information provision | 1 | 2 | 3 | 4 | 5 |
| Cost paid | 1 | 2 | 3 | 4 | 5 |
| Confidentiality of provider | 1 | 2 | 3 | 4 | 5 |
| Drug and supplies availability | 1 | 2 | 3 | 4 | 5 |
| Privacy level | 1 | 2 | 3 | 4 | 5 |
| Respect and courtesy of staff | 1 | 2 | 3 | 4 | 5 |
| Examination room cleanliness | 1 | 2 | 3 | 4 | 5 |
| Waiting area cleanliness | 1 | 2 | 3 | 4 | 5 |
| Waiting time | 1 | 2 | 3 | 4 | 5 |
| Overall cleanliness of facility | 1 | 2 | 3 | 4 | 5 |
